# A case study of quark-gluon discrimination at NNLL$'$ in comparison to parton showers

**DOI:** 10.1140/epjc/s10052-017-5365-9

**Published:** 2017-11-16

**Authors:** Jonathan Mo, Frank J. Tackmann, Wouter J. Waalewijn

**Affiliations:** 1 Institute for Theoretical Physics Amsterdam and Delta Institute for Theoretical Physics, University of Amsterdam, Science Park 904, 1098 XH Amsterdam, The Netherlands; 2 Nikhef, Theory Group, Science Park 105, 1098 XG Amsterdam, The Netherlands; 3 Theory Group, Deutsches Elektronen-Synchrotron (DESY), 22607 Hamburg, Germany

## Abstract

Predictions for our ability to distinguish quark and gluon jets vary by more than a factor of two between different parton showers. We study this problem using analytic resummed predictions for the thrust event shape up to NNLL$'$ using $e^+e^- \rightarrow Z \rightarrow q \bar{q}$ and $e^+e^- \rightarrow H \rightarrow gg$ as proxies for quark and gluon jets. We account for hadronization effects through a nonperturbative shape function, and include an estimate of both perturbative and hadronization uncertainties. In contrast to previous studies, we find reasonable agreement between our results and predictions from both Pythia and Herwig parton showers. We find that this is due to a noticeable improvement in the description of gluon jets in the newest Herwig 7.1 compared to previous versions.

## Introduction

The reliable discrimination between quark-initiated and gluon-initiated jets is a key goal of jet substructure methods [1–4]. It would provide a direct handle to distinguish hard processes that lead to the same number but different types of jets in the final state. A representative example is the search for new physics, where the signal processes typically produce quark jets, while QCD backgrounds predominantly involve gluon jets from gluon radiation.

Jet substructure observables for quark-gluon discrimination have been studied extensively using both parton showers and analytic calculations [5–14]. Much effort has been dedicated to identifying the most promising observables to achieve this goal. However, it has been known for a while that the discrimination power one obtains differs a lot between different parton shower predictions. A detailed study has been carried out in Refs. [10, 14]. It uses the classifier

1 $$\begin{aligned} \Delta = \frac{1}{2} \int \! \mathrm {d}\lambda \, \frac{\big [p_q(\lambda ) - p_g(\lambda )\big ]^2}{p_q(\lambda )+p_g(\lambda )} \end{aligned}$$

to quantify the differences between the normalized quark and gluon distributions $p_{q,g}$ for an observable $\lambda$, providing a measure of the quark-gluon separation. The study found that the various parton showers agree well in their predictions for quark jets, which is not surprising since much information on the shape of quark jets is available from LEP data. On the other hand, there is still very little information on gluon jets available, and correspondingly the study identified the substantially different predictions for gluon jets as the main culprit.

Parton showers are formally only accurate to (next-to-) leading logarithmic order and do not provide an estimate of their intrinsic perturbative (resummation) uncertainties. Thus, it is not clear to what extent the observed differences are a reflection of (and thus consistent within) the inherent uncertainties, or whether only some of the parton showers obtain correct predictions.

In this paper, we address this issue by considering the thrust event shape for which we are able to obtain precise theoretical predictions from analytic higher-order resummed calculations, which can be used as a benchmark for parton-shower predictions. An extensive survey of parton-shower predictions as carried out in Refs. [10, 14] is beyond our scope here. We will instead restrict ourselves to Pythia  [15] and Herwig  [16], as they represent the opposite extremes in the results of Refs. [10, 14].

Thrust has been calculated to (next-to-)next-to-next-to-leading logarithmic ((N)NNLL) accuracy for quark jets produced in $e^+e^- \rightarrow q\bar{q}$ collisions [17, 18]. Here, we also obtain new predictions at NNLL$'$ for gluonic thrust using the toy process $e^+e^-\rightarrow H \rightarrow gg$, from which we can then calculate the quark-gluon classifier separation at NNLL$'$.^1^ Thrust is defined as

2 $$\begin{aligned} T = \max {}_{\hat{t}}\; \frac{\sum _i |\hat{t} \!\cdot \!{\mathbf {p}}_i|}{\sum _i |{\mathbf {p}}_i|},\quad \tau = 1 - T, \end{aligned}$$

where the sum over *i* runs over all final-state particles. For $\tau \ll 1$, the final state consists of two back-to-back jets initiated by the back-to-back quarks or gluons produced in the hard interaction. The different radiation patterns in these jets is probed by $\tau$, since in this limit

3 $$\begin{aligned} \tau = \frac{M_1^2 + M_2^2}{Q^2}, \end{aligned}$$

where $M_{1,2}$ are the invariant masses of the two (hemisphere) jets and *Q* is the invariant mass of the collision. Thrust corresponds closely to the generalized angularity $(\kappa ,\beta ) = (1,2)$, which is the jet mass and was one of the benchmark observables considered in Refs. [10, 14]. While we consider hemisphere jets, the jet angularities only sum over particles within a certain jet radius around the thrust axis. However, for our purposes of providing a benchmark for the radiation pattern produced by the parton showers this difference in the jet size is not relevant.

Our numerical results include resummation up to NNLL$'$ resummation and include nonperturbative hadronization corrections through a shape function [19–22]. We assess the perturbative uncertainty through appropriate variations of the profile scales [18, 22], and the nonperturbative uncertainty by varying the nonperturbative parameter $\Omega$, which quantifies the leading nonperturbative corrections.

**Fig. 1:**
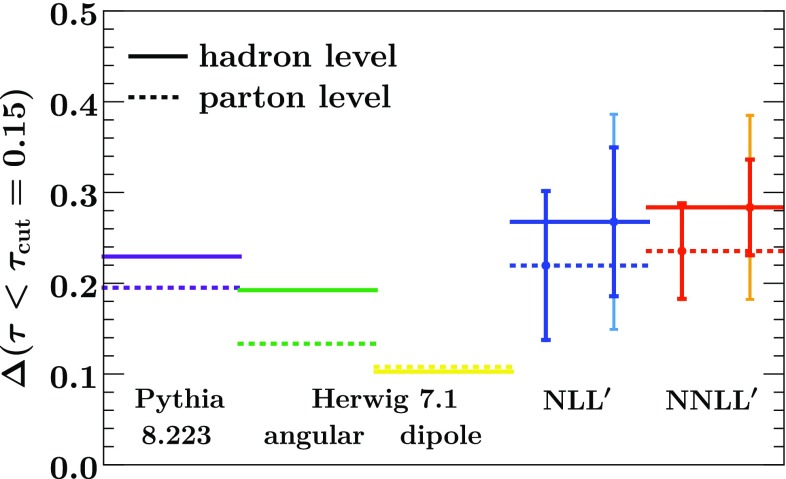
The quark-gluon classifier separation $\Delta$ for $\tau < 0.15$ from Pythia 8.223 (violet), Herwig 7.1 angular-ordered shower (green) and dipole shower (yellow) compared to analytic resummation at NLL$'$ (blue) and NNLL$'$ (red). The results at parton and hadron level are shown in dotted and solid, respectively. The uncertainty bars on the resummed results show the perturbative uncertainty and also the sum of perturbative and hadronization uncertainties (lighter outer bars at hadron level)

Figure 1 shows the classifier separation for quark-gluon discrimination in Eq. (1) at parton and hadron level obtained from our analytic predictions, compared to Pythia 8.223 [15] and Herwig 7.1 [16]. Our resummed results are shown at NLL$'$ and NNLL$'$, and include an estimate of the perturbative and hadronization uncertainty. As we do not combine our NNLL$'$ prediction with the full fixed-order NNLO result, which would become relevant at large $\tau$, we restrict the integration range here to $\tau < 0.15$. Both Pythia ’s parton shower and Herwig ’s default angular-ordered shower are consistent with our results. We observe that the tension between these two showers is much reduced here compared to what was found in Refs. [10, 14]. As we will see later, this is due to an improved description of gluon jets in Herwig 7.1 compared to earlier versions. Specifically, the parton shower now preserves the virtuality rather than the transverse momentum after multiple emissions, and has been tuned to gluon data for the first time [23]. For comparison, we also include results obtained using Herwig ’s dipole shower, which still gives substantially lower predictions compared to the others.

The outline of this paper is as follows: In Sect. 2 we present the details of our calculation. Many of the ingredients can be found in the literature but are reproduced here (and in appendices) to make the paper self-contained. We present numerical results in Sect. 3 for the thrust distribution of quark and gluons jets, as well as the classifier separation calculated from it, and performing comparisons to Pythia and Herwig. In Sect. 4 we conclude.

## Calculation

The cross section for thrust factorizes [19, 24–26]

4 $$\begin{aligned} \frac{\mathrm {d}\sigma _i}{\mathrm {d}\tau }= & {} \sigma _{i,0}\, |C_i(Q,\mu )|^2 \int \! \mathrm {d}s_1\, J_i(s_1,\mu ) \int \! \mathrm {d}s_2\, J_i(s_2,\mu ) \nonumber \\&\times \int \! \mathrm {d}k\, S_i(k,\mu )\, \delta \left( \tau - \frac{s_1 + s_2}{Q^2} - \frac{k}{Q}\right) + \frac{\mathrm {d}\sigma _i^\mathrm{nons}}{\mathrm {d}\tau },\nonumber \\ \end{aligned}$$

where the label $i=q$ corresponds to the hard process $Z \rightarrow q \bar{q}$ and $i=g$ corresponds to $H \rightarrow gg$. The Born cross section is denoted by $\sigma _{i,0}$, with hard virtual corrections contained in the hard Wilson coefficient $C_i$. The jet functions $J_i$ describes the invariant masses $s_{1,2}$ of the energetic (collinear) radiation in the jets. The soft function $S_i$ encodes the contribution *k* of soft radiation to the thrust measurement. Contributions that do not factorize in this manner are suppressed by relative $\mathcal {O}(\tau )$ and are contained in the nonsingular cross section $\mathrm {d}\sigma _i^\mathrm{nons}/\mathrm {d}\tau$.

### Resummation

For $\tau \ll 1$ the thrust spectrum contains large logarithms of $\tau$, that we resum by utilizing the renormalization group evolution that follows from the factorization in Eq. (4). This is accomplished by evaluating $C_i$, $J_i$, and $S_i$ at their natural scales

5 $$\begin{aligned} \mu _C \simeq Q, \quad \mu _J \simeq \sqrt{\tau Q}, \quad \mu _S \simeq \tau Q, \end{aligned}$$

where they each do not contain large logarithms, and evolving them to a common (and arbitrary) scale $\mu$. The precise resummation scales and their variations used in our numerical results are given in Eq. (11).

The renormalization group equations of the hard, jet, and soft functions are given by

6 $$\begin{aligned} \mu \frac{\mathrm {d}}{\mathrm {d}\mu }\, C_i(Q, \mu )= & {} \gamma _C^i(Q, \mu )\, C_i(Q, \mu ), \\ \gamma _C^i(Q, \mu )= & {} \Gamma _\mathrm{cusp}^i[\alpha _s(\mu )] \ln \frac{Q^2}{\mu ^2} + 2\gamma _C^i[\alpha _s(\mu )],\nonumber \\ \mu \frac{\mathrm {d}}{\mathrm {d}\mu }\, J_i(s, \mu )= & {} \int \! \mathrm {d}s'\, \gamma _J^i(s-s',\mu )\, J_i(s', \mu ),\nonumber \\ \gamma _J^i(s, \mu )= & {} -2 \Gamma ^i_{\mathrm{cusp}}[\alpha _s(\mu )]\,\frac{1}{\mu ^2} \biggl [\frac{\mu ^2}{s}\biggr ]_+\nonumber \\&+ \gamma _J^i[\alpha _s(\mu )] \delta (s),\nonumber \\ \mu \frac{\mathrm {d}}{\mathrm {d}\mu }\, S_i(k, \mu )= & {} \int \! \mathrm {d}k'\, \gamma _S^i(k\! - k', \mu )\, S_i(k', \mu ),\nonumber \\ \gamma _S^i(k, \mu )= & {} 4\,\Gamma _\mathrm{cusp}^i[\alpha _s(\mu )]\, \frac{1}{\mu } \biggl [\frac{\mu }{k}\biggr ]_+\nonumber \\&+ \gamma _S^i[\alpha _s(\mu )]\, \delta (k),\nonumber \end{aligned}$$

and involve the cusp anomalous dimension $\Gamma _\mathrm{cusp}^i(\alpha _s)$ [27] and a noncusp term $\gamma _{C,J,S}^i(\alpha _s)$. (The factor of 2 in front of $\gamma _C^i(\alpha _s)$ is included to be consistent with our conventions in e.g. Ref. [28].) The $\mu$ independence of the cross section in Eq. (4) implies the consistency condition

7 $$\begin{aligned} 4\gamma _C^i(\alpha _s) + 2\gamma _J^i(\alpha _s) + \gamma _S^i(\alpha _s) = 0. \end{aligned}$$

We employ analytic solutions to the RG equations, which for the jet and soft function follow from Refs. [29–31]. For our implementation we use the results for the RG solution and plus-function algebra derived in Ref. [22].

The ingredients that enter the cross section at various orders of resummed perturbation theory are summarized in Table 1. Our best predictions are at NNLL$'$ order, which is closer to NNNLL than NNLL, as the inclusion of the two-loop fixed-order ingredients has a larger effect than the three-loop non-cusp and four-loop cusp anomalous dimension. Our NNLL$'$ predictions require the two-loop hard function [32–39], jet function [40, 41], and soft function [42, 43]. The RG evolution involves the three-loop QCD beta function [44, 45], three-loop cusp anomalous dimension [46] and two-loop non-cusp anomalous dimensions [35, 36, 36, 47]. All necessary expressions are collected in the appendices. In our numerical analysis we take $\alpha _s(m_Z) = 0.118$.

### Nonsingular corrections

To obtain a reliable description of the thrust spectrum for large values of $\tau$ we also need to include the nonsingular $\mathrm {d}\sigma _i^\mathrm{nons}/\mathrm {d}\tau$ in Eq. (4). These are obtained from the full $\mathcal {O}(\alpha _s)$ expressions

8 $$\begin{aligned} \frac{\mathrm {d}\sigma _q}{\mathrm {d}\tau }= & {} \sigma _{q,0}\, \frac{\alpha _s C_F}{2\pi }\, \frac{1}{\tau (\tau -1)}\biggl [3 - 9 \tau - 3 \tau ^2 + 9 \tau ^3 \nonumber \\&-\,(4-6\tau +6\tau ^2) \ln \frac{1- 2\tau }{\tau } \biggr ], \nonumber \\ \frac{\mathrm {d}\sigma _g}{\mathrm {d}\tau }= & {} \sigma _{g,0}\,\frac{\alpha _s}{2\pi } \biggl \{C_A\, \frac{1}{3\tau (\tau -1)}\biggl [11 - 68\tau + 144\tau ^2 - 132\tau ^3 \nonumber \\&+\,45\tau ^4 - 12(1 - 2\tau + 3\tau ^2 - 2\tau ^3 + \tau ^4)\ln \frac{1-2\tau }{\tau } \biggr ]\nonumber \\&+\, T_F n_f\, \frac{2}{3\tau }\biggl [2 - 21\tau + 60\tau ^2 -45\tau ^3 \nonumber \\&+\, 6\tau (1 - 2\tau + 2\tau ^2)\ln \frac{1-2\tau }{\tau } \biggr ] \biggr \}, \end{aligned}$$

and subtracting the terms that are singular in the $\tau \rightarrow 0$ limit, which are contained in the NLL$'$ resummed result. Adding the $\mathcal {O}(\alpha _s)$ nonsingular corrections to the NLL$'$ resummed cross section then yields the final matched NLL$'+$NLO result. The above result for the quark case has been known for a long time [48]. The gluon result was obtained by squaring and summing the helicity amplitudes in Ref. [49] and performing the required phase-space integrations to project onto the $\tau$ spectrum. At NNLL$'$ we would also need the full $\mathcal {O}(\alpha _s^2)$ terms to obtain the matched NNLL$'+$NNLO result, so we restrict ourselves to small $\tau < 0.15$ in this case, such that we can neglect the nonsingular corrections.

**Table 1:** Perturbative ingredients at different orders in resummed perturbation theory

	$C_i, J_i, S_i$	$\gamma _C^i, \gamma _J^i, \gamma _S^i$	$\Gamma _\mathrm{cusp}, \beta$
LL	0-loop	–	1-loop
NLL	0-loop	1-loop	2-loop
NLL$'$	1-loop	1-loop	2-loop
NNLL	1-loop	2-loop	3-loop
NNLL$'$	2-loop	2-loop	3-loop
NNNLL	2-loop	3-loop	4-loop

### Hadronization effects

The soft function in the factorization theorem in Eq. (4) accounts for both perturbative soft radiation and nonperturbative hadronization effects. The hadronization effects can be taken into account by factorizing the full soft function as [19, 21, 22]

9 $$\begin{aligned} S_i(k,\mu ) = \int \! \mathrm {d}k'\, S_i^\mathrm{pert}(k-k',\mu ) F_i(k'), \end{aligned}$$

where $S_i^\mathrm{pert}(k,\mu )$ contains the perturbative corrections and $F_i(k)$ is a nonperturbative shape function encoding hadronization effects. This treatment is known to provide an excellent description of hadronization effects in *B*-meson decays [50] and $e^+e^-$ event shapes [18]. It has furthermore been successfully utilized for quark and gluon jet mass spectra in hadron collisions [51].

The shape function $F_i(k)$ is normalized to unity and has typical support for $k \sim \Lambda _\mathrm {QCD}$. It should vanish at $k = 0$ and fall off exponentially for $k\rightarrow \infty$. We use a simple ansatz that satisfies these basic criteria [51]

10 $$\begin{aligned} F_i(k') = \frac{k'}{\Omega _i^2} e^{-k'/ \Omega _i}. \end{aligned}$$

The parameter $\Omega _i$ captures the leading nonperturbative correction in the tail of the distribution, where it leads to a shift $\tau \rightarrow \tau + 2\Omega _i/Q$. We take $\Omega _q = 0.4$ [18] and assume Casimir scaling, $\Omega _g = \Omega _q C_A/C_F$. As an estimate of the nonperturbative uncertainty we vary $\Omega _q$ and $\Omega _g$ over a large range as discussed above Eq. (17). In the peak of the distribution in principle the full functional form of $F_i(k)$ enters. However, given the large uncertainties for $\Omega _i$ we currently include, the precise functional form of $F_i$ is not yet of practical importance.

### Estimation of uncertainties

The canonical scales in Eq. (5) do not properly take into account the transition from the resummation region into the fixed-order region where $\tau$ is no longer small, or into the nonperturbative region for $\tau \lesssim \Lambda _\mathrm {QCD}/Q$. A smooth transition between these different regimes is accomplished using profile scales [18, 22].

For the choice of profiles scales and the estimation of perturbative uncertainties through their variations we follow the approach of Ref. [52] adapted to the thrust-like resummation as in Ref. [53]. The central values for the profile scales are taken as

11 $$\begin{aligned}&\mu _H = \mu , \quad \mu _S(\tau ) = \mu f_\mathrm{run}(\tau ), \quad \mu _J(\tau ) = \sqrt{\mu _S(\tau ) \mu }, \nonumber \\&f_\mathrm{run} (\tau ) = \left\{ \begin{array}{ll} \tau _0(1+ \frac{\tau ^2}{(2\tau _0)^2}) &{}\quad \tau \le 2 \tau _0\\ \tau &{}\quad 2\tau _0 \le \tau \le \tau _1 \\ \tau +\frac{(2-\tau _2-\tau _3)(\tau -\tau _1)^2}{2(\tau _2-\tau _1)(\tau _3-\tau _1)} &{}\quad \tau _1 \le \tau \le \tau _2\\ 1 -\frac{(2-\tau _1-\tau _2)(\tau -\tau _3)^2}{2(\tau _3-\tau _1)(\tau _3-\tau _2)} &{}\quad \tau _2 \le \tau \le \tau _3\\ 1 &{}\quad \tau _3 \le \tau \end{array} \right. \end{aligned}$$

Here, $\tau _0$ determines the boundary between the resummation and nonperturbative region, where the jet and soft scales approach $\sqrt{\tau _0}Q$ and $\tau _0 Q$ respectively. We choose $\tau _0 = 3 \mathrm {GeV}/ Q$, so that $\mu _J$, $\mu _S$ are always greater than $\Lambda _{\mathrm {QCD}}$. From $\tau _0$ onwards we have the canonical resummation scales in Eq. (5) up to $\tau _1 = 0.1$, where the different scales are still well separated. Then we smoothly turn the resummation off by letting $f_{\mathrm {run}}(\tau )$ go to 1. The resummation is completely turned off at $\tau _3 = 1/3$, where the singular and nonsingular contributions start to cancel each other exactly at $\mathcal {O}(\alpha _s)$. The central curve of our prediction corresponds to

12 $$\begin{aligned}&\mu = Q, \quad \tau _0 = \frac{3 \mathrm {GeV}}{Q}, \quad \tau _1 = 0.1, \nonumber \\&\quad \tau _2 = \frac{\tau _1 + \tau _3}{2}, \quad \tau _3 = \frac{1}{3}. \end{aligned}$$

**Fig. 2:**
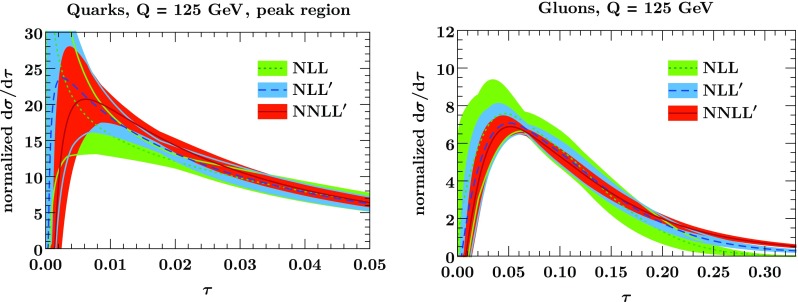
The normalized thrust spectrum at NLL (green), NLL$'$ (blue), and NNLL$'$ (orange) for quarks (left panel) and gluons (right panel). Since the quark distribution on the left is peaked at small $\tau$, we restrict the plot range to $\tau <0.05$. The bands indicate the perturbative uncertainty at each order, obtained using Eq. (13)

The perturbative uncertainty is obtained as the quadratic sum of a fixed-order and a resummation contribution,

13 $$\begin{aligned} \delta _\mathrm{pert} = \sqrt{\delta _\mathrm{FO}^2 + \delta _\mathrm{resum}^2}. \end{aligned}$$

The fixed-order uncertainty is estimated by the maximum observed deviation from varying the parameter $\mu$ in Eq. (11) by a factor of two,

14 $$\begin{aligned} \delta _\mathrm{FO} (\tau ) = \max _{\mu = \{2Q, Q/2\}} \bigg |\frac{\mathrm {d}\sigma }{\mathrm {d}\tau } - \frac{\mathrm {d}\sigma _\mathrm{central}}{\mathrm {d}\tau } \bigg |. \end{aligned}$$

The resummation uncertainty is estimated by varying $\mu _{J,S}$ by [53]

15 $$\begin{aligned} \mu ^\mathrm{vary}_S(\tau ,\alpha )= & {} f_\mathrm{vary}^\alpha (\tau )\, \mu _S(\tau ), \nonumber \\ \mu ^\mathrm{vary}_J(\tau ,\alpha ,\beta )= & {} \mu ^\mathrm{vary}_S(\tau ,\alpha )^{1/2-\beta } \mu ^{1/2+\beta },\nonumber \\ f_\mathrm{vary}(\tau )= & {} \left\{ \begin{array}{ll} 2(1-\tau ^2/\tau _3^2) &{}\quad \tau \le \tau _3/2\\ 1 + 2(1-\tau /\tau _3)^2 &{}\quad \tau _3/2 \le \tau \le \tau _3\\ 1 &{}\quad \tau _3 \le \tau \end{array} \right. \end{aligned}$$

and taking the maximum absolute deviation among all variations

16 $$\begin{aligned} \delta _\mathrm{resum} (\tau ) = \max _{(\alpha ,\beta )} \bigg |\frac{\mathrm {d}\sigma }{\mathrm {d}\tau } - \frac{\mathrm {d}\sigma _\mathrm{central}}{\mathrm {d}\tau } \bigg |, \end{aligned}$$

with $(\alpha ,\beta ) \in \{(1,0),(-1,0),(0,1/6), (0,- 1/6)\}$. Furthermore, we vary the transition points $\tau _0$ and $\tau _1$ of the resummation region by $\pm \, 25\%$. These variations however have a much smaller effect than the $\alpha ,\beta$ variations, and their effect on the final resummation uncertainty is almost negligible.

To account for hadronization uncertainties, we separately vary $\Omega _q$ by $\pm \, 50\%$, $\Omega _g$ by $\pm \, 50\%$, and simultaneously vary $\Omega _q$ and $\Omega _g$ by $\pm \,75\%$. The hadronization uncertainty $\delta _\mathrm{nonp}$ is then taken as the maximum deviation under these variations. It is treated as a separate uncertainty source uncorrelated from the perturbative uncertainty, with the total uncertainty given by their quadratic sum,

17 $$\begin{aligned} \delta = \sqrt{\delta _\mathrm{pert}^2 + \delta _\mathrm{nonp}^2}. \end{aligned}$$

We follow a similar procedure to assess the uncertainty on the classifier separation. However, we do not vary the quark distribution and gluon distribution simultaneously, as varying them in opposite directions would lead to an unrealistic inflation of the uncertainty. Instead, we obtain the uncertainty on the classifier separation by taking the central quark result and varying the gluon distribution, and vice versa. This amounts to treating the perturbative uncertainties in the quark and gluon distributions as uncorrelated sources of uncertainties.

**Fig. 3:**
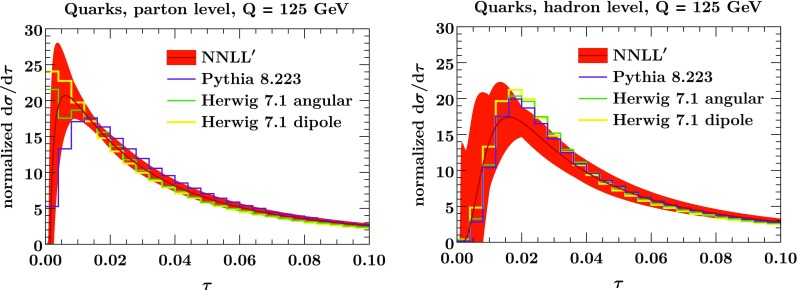
The normalized thrust spectrum for quarks at NNLL$'$ (orange band) compared to Pythia (violet) and Herwig ’s angular-ordered (green) and dipole shower (yellow) at parton level (left panel) and hadron level (right panel). The band in the left panel shows the perturbative uncertainty in Eq. (13). In the right panel, it shows the sum of perturbative and nonperturbative uncertainties as in Eq. (17)

**Fig. 4:**
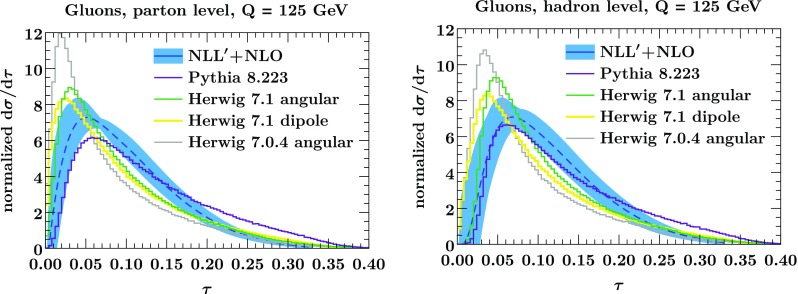
The normalized thrust spectrum for gluons at NLL$'+$NLO (blue band) compared to Pythia (violet) and Herwig ’s angular-ordered (green) and dipole shower (yellow) at parton level (left panel) and hadron level (right panel). The band in the left panel shows the perturbative uncertainty in Eq. (13). In the right panel, it shows the sum of perturbative and nonperturbative uncertainties as in Eq. (17). The result from the angular-ordered shower in Herwig 7.0.4 is shown in light gray, which differs significantly from the resummed results, highlighting the noticeable improvement in Herwig 7.1

## Results

We now present our numerical results and compare these to Pythia and Herwig. We restrict ourselves to normalized distributions, as these are the input entering in the classifier separation in Eq. (1).

Figure 2 shows the thrust spectrum for quarks and gluons at various orders in resummed perturbation theory. The bands show the perturbative uncertainty, obtained using the procedure described in Sect. 2.4. The overlapping uncertainty bands suggest that our uncertainty estimate is reasonable, and the reduction of the uncertainty at higher orders indicates the convergence of our resummed predictions. This is not true for large values of $\tau$, because we did not include the nonsingular corrections $\mathrm {d}\sigma ^\mathrm{nons}/\mathrm {d}\tau$ that are important in this region.

In Figs. 3 and 4 we compare our predictions for quarks and gluons at parton and hadron level to Pythia and Herwig. Note that the peak of the quark distribution is in the nonperturbative regime $\tau Q \lesssim \Lambda _\mathrm {QCD}$. Therefore we restrict to $\tau < 0.1$ when considering the quark distributions in Fig. 3, allowing the use of the NNLL$'$ result. On the other hand, the gluon distribution peaks at much higher values, and so we consider the gluon distribution over the full $\tau$ range using the matched result at NLL$'$+NLO.

For quarks at parton level, shown in the left panel of Fig. 3, both Pythia and Herwig agree well with the resummed result and also with each other. The only exception is in the nonperturbative regime at very small $\tau$, where the comparison of parton-level predictions is not very meaningful. At the hadron level (right panel of Fig. 3) we also include the nonperturbative uncertainty in our band, and our predictions agree well with Pythia and Herwig. Note that Pythia and Herwig at hadron level agree with each other even better than at parton level. This is of course not surprising, as their hadronization models have been tuned to the same LEP data. The differences seen at parton level are likely due to a higher shower cutoff scale in Herwig (which would also explain the events with $\tau =0$), and is compensated for by the hadronization [14].

We now turn to the results for gluons shown in Fig. 4. Here, there differences between Pythia and Herwig are much larger at both parton and hadron level. At parton level and small values of $\tau$, the Herwig 7.1 and Pythia predictions touch opposite sides of the uncertainty band of the NLL$'$+NLO result. Thus, although the differences in the parton shower results are clearly sizeable, they might still be considered to be within their intrinsic uncertainties, also since the formal accuracy of the showers is less than that of the NLL$'+$NLO result. For large values beyond $\tau > 0.2$ there are differences between Pythia and our result. However, this region is not described by the resummation but the fixed-order calculation. At NLO there are only three partons, so $\tau \le 1/3$. Although Pythia produces events with $\tau >1/3$, it does not do so with any formal accuracy, since the parton shower is built from collinear/soft limits of QCD which do not apply here.

For gluons at hadron level, Pythia agrees well with our result. The agreement for Herwig 7.1 is less good, though the differences are not that large either. However, we see that the angular ordered shower from Herwig 7.0.4 shown by the gray lines shows clear discrepancies from our predictions. (It also yields similarly large differences between Herwig and Pythia for the quark-gluon separation as observed for Herwig 2.7.1 in Refs. [10, 14].) This highlights the substantial improvement in the description of gluon jets in the latest version of Herwig.

**Fig. 5:**
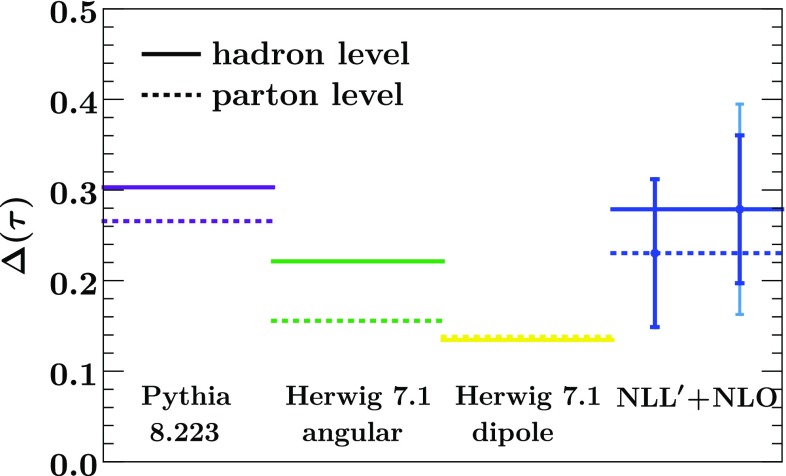
Analogous to Fig. 1 but without a cut on $\tau$

Finally, in Fig. 5 we show the classifier separation at NLL$'$+NLO compared to Pythia and Herwig at parton and hadron level. This is similar to Fig. 1, but we do not impose a cut on thrust and therefore omit the NNLL$'$ result. The perturbative uncertainty $\delta _\mathrm{pert}$ is shown, as well as the total uncertainty. Both Pythia and Herwig agree with our results within uncertainties. They differ from each other more than in Fig. 1, which is due to the relatively large differences in the gluon distribution at larger $\tau$. Herwig predicts a lower classifier separation $\Delta$, because its gluon distribution is peaked at smaller values of $\tau$ and thus closer to the quark distribution. As in Fig. 1, this is most pronounced for the Herwig dipole shower, which has the gluon distribution with the lowest peak and as a result gives the lowest $\Delta$.

Finally, it is worth noting that the resummation and hadronization uncertainties on the classifier separation are of similar size. Thus at higher orders the hadronization uncertainty currently becomes the limiting factor, as can be seen in the NNLL$'$ results in Fig. 1. This is of course also due to our rather generous variations for the hadronization parameter $\Omega _i$. This situation can be improved by using a more refined treatment than carried out here, including renormalon subtractions and performing a fit to LEP data as done in Ref. [18], which yields a much more precise determination of $\Omega _q$. However, one would then also have to perform a more careful treatment of the full shape function in the nonperturbative peak region of the quark distribution, for example using the methods of Refs. [22, 50].

## Conclusions

Large differences have been observed between parton showers in their prediction for our ability to discriminate quark jets from gluon jets. This inspired us to consider the thrust event shape, which can be calculated very precisely, obtaining a sample of quark jets from $Z \rightarrow q \bar{q}$ and gluon jets from $H \rightarrow gg$. We compared our analytic results up to NNLL$'$ to Pythia and Herwig, which represented the two opposite extremes in an earlier study [10, 14]. Our results are consistent with both Pythia and Herwig, though closest to Pythia. This is due to the improved description of gluon jets in the most recent Herwig release, while the previous Herwig 7.0.4 showed substantial differences in the gluon distribution. Resummed predictions, like those obtained here, can thus serve as an important standard candle for parton showers. The perturbative uncertainties can be reduced further by going to higher orders. At NNLL$'$ the uncertainty from nonperturbative effects currently constitutes the limiting factor in the resummed results, which can be improved in the future with a more refined treatment of nonperturbative corrections.

## Appendix A: Anomalous dimensions

Expanding the beta function and anomalous dimensions in powers of $\alpha _s$,

A1 $$\begin{aligned} \beta (\alpha _s)= & {} - 2 \alpha _s \sum _{n=0}^\infty \beta _n\Bigl (\frac{\alpha _s}{4\pi }\Bigr )^{n+1}, \nonumber \\ \Gamma ^i_\mathrm{cusp}(\alpha _s)= & {} \sum _{n=0}^\infty \Gamma ^i_n \Bigl (\frac{\alpha _s}{4\pi }\Bigr )^{n+1}, \nonumber \\ \gamma ^i(\alpha _s)= & {} \sum _{n=0}^\infty \gamma ^i_{n} \Bigl (\frac{\alpha _s}{4\pi }\Bigr )^{n+1}, \end{aligned}$$

the coefficients are given by

A2 $$\begin{aligned} \beta _0= & {} \frac{11}{3}\,C_A -\frac{4}{3}\,T_F\,n_f,\nonumber \\ \beta _1= & {} \frac{34}{3}\,C_A^2 - \left( \frac{20}{3}\,C_A\, + 4 C_F\right) \, T_F\,n_f, \nonumber \\ \beta _2= & {} \frac{2857}{54}\,C_A^3 \nonumber \\&+ \left( C_F^2 - \frac{205}{18}\,C_F C_A - \frac{1415}{54}\,C_A^2 \right) \, 2T_F\,n_f\nonumber \\&+ \left( \frac{11}{9}\, C_F + \frac{79}{54}\, C_A \right) \, 4T_F^2\,n_f^2,\nonumber \\ \Gamma ^q_0= & {} 4C_F,\nonumber \\ \Gamma ^q_1= & {} 4C_F \left[ \left( \frac{67}{9} -\frac{\pi ^2}{3} \right) \,C_A - \frac{20}{9}\,T_F\, n_f \right] ,\nonumber \\ \Gamma ^q_2= & {} 4C_F \left[ \left( \frac{245}{6} - \frac{134 \pi ^2}{27} + \frac{11 \pi ^4}{45} + \frac{22 \zeta _3}{3}\right) C_A^2 \right. \nonumber \\&- \left( \frac{418}{27} - \frac{40 \pi ^2}{27} + \frac{56 \zeta _3}{3} \right) C_A\, T_F\,n_f \nonumber \\&\left. -\left( \frac{55}{3} - 16 \zeta _3 \right) C_F\, T_F\,n_f - \frac{16}{27}\,T_F^2\, n_f^2 \right] ,\nonumber \\ \gamma ^q_{C\,0}= & {} -3 C_F,\nonumber \\ \gamma ^q_{C\,1}= & {} - C_F \left[ \left( \frac{41}{9} - 26 \zeta _3\right) C_A + \left( \frac{3}{2} - 2 \pi ^2 + 24 \zeta _3\right) C_F \right. \nonumber \\&\left. +\left( \frac{65}{18} + \frac{\pi ^2}{2} \right) \beta _0 \right] , \nonumber \\ \gamma _{S\,0}^q= & {} 0,\nonumber \\ \gamma _{S\,1}^q= & {} C_F \left[ \left( -\frac{128}{9} + 56 \zeta _3\right) C_A + \left( -\frac{112}{9} + \frac{2\pi ^2}{3} \right) \beta _0 \right] ,\nonumber \\ \gamma _{C\,0}^g= & {} - \beta _0,\nonumber \\ \gamma _{C\,1}^g= & {} \left( -\frac{59}{9} + 2\zeta _3\right) C_A^2 + \left( -\frac{19}{9}+\frac{\pi ^2}{6} \right) C_A\, \beta _0 - \beta _1.\nonumber \\ \end{aligned}$$

The expressions for $\Gamma ^g$ and $\gamma _S^g$ are omitted, as they can be obtained from Casimir scaling

A3 $$\begin{aligned} \Gamma _n^g = \frac{C_A}{C_F}\,\Gamma _n^q \,, \quad \gamma _{S\,n}^g = \frac{C_A}{C_F}\,\gamma _{S\,n}^q. \end{aligned}$$

The coefficients $\gamma _J^i$ of the non-cusp anomalous dimension of the jet function follow from Eq. (7).

## Appendix B: Fixed-Order Ingredients

The form of the Wilson coefficient, jet function and soft function is highly constrained by the anomalous dimensions in Eq. (6),

B1 $$\begin{aligned}&C_q(Q,\mu ) = 1 + \frac{\alpha _s(\mu )}{4\pi } \left[ - \frac{\Gamma _0^q}{4} \ln ^2 \left( \frac{-q^2-\mathrm {i}0}{\mu ^2}\right) \right. \nonumber \\&\quad \left. -\, \gamma _{C\,0}^q \ln \left( \frac{-q^2-\mathrm {i}0}{\mu ^2}\right) + c_0^q \right] \nonumber \\&\quad + \left( \frac{\alpha _s(\mu )}{4\pi }\right) ^2 \left[ \frac{(\Gamma _0^q)^2}{32} \ln ^4 \left( \frac{-q^2-\mathrm {i}0}{\mu ^2}\right) \right. \nonumber \\&\quad +\, \frac{\Gamma _0^q(3\gamma _{C\,0}^q + \beta _0)}{12} \ln ^3 \left( \frac{-q^2-\mathrm {i}0}{\mu ^2}\right) \nonumber \\&\quad +\, \frac{2(\gamma _{C\,0}^q)^2 + 2 \beta _0 \gamma _{C\,0}^q - \Gamma _1^q - \Gamma _0^q c_0^q}{4} \ln ^2 \left( \frac{-q^2-\mathrm {i}0}{\mu ^2}\right) \nonumber \\&\quad \left. -\,(\gamma _{C\,1}^q + \gamma _{C\,0}^q c_0^q + \beta _0 c_0^q) \ln \left( \frac{-q^2-\mathrm {i}0}{\mu ^2}\right) + c_1^q \right] ,\nonumber \\&C_g(Q,\mu ) = \alpha _s\left\{ 1 + \frac{\alpha _s(\mu )}{4\pi } \left[ - \frac{\Gamma _0^g}{4} \ln ^2 \left( \frac{-q^2-\mathrm {i}0}{\mu ^2}\right) \right. \right. \nonumber \\&\quad \left. -\, (\gamma _{C\,0}^g + \beta _0) \ln \left( \frac{-q^2-\mathrm {i}0}{\mu ^2}\right) + c_0^g \right] \nonumber \\&\quad + \left( \frac{\alpha _s(\mu )}{4\pi }\right) ^2 \left[ \frac{(\Gamma _0^g)^2}{32} \ln ^4 \left( \frac{-q^2-\mathrm {i}0}{\mu ^2}\right) \right. \nonumber \\&\quad +\,\frac{\Gamma _0^g(3\gamma _{C\,0}^g + 4\beta _0)}{12} \ln ^3 \left( \frac{-q^2-\mathrm {i}0}{\mu ^2}\right) \nonumber \\&\quad +\, \frac{2(\gamma _{C\,0}^g)^2 + 6\beta _0 \gamma _{C\,0}^g + 4\beta _0^2 - \Gamma _1^g - \Gamma _0^g c_0^g}{4} \ln ^2 \left( \frac{-q^2-\mathrm {i}0}{\mu ^2}\right) \nonumber \\&\quad \left. -\, (\gamma _{C\,1}^g + \gamma _{C\,0}^g c_0^g + 2\beta _0 c_0^g + \beta _1) \ln \left( \frac{-q^2-\mathrm {i}0}{\mu ^2}\right) + c_1^g \right\} , \nonumber \\&J_i(s,\mu ) = \delta (s) + \frac{\alpha _s(\mu )}{4\pi } \left[ \frac{\Gamma _0^i}{\mu ^2} \left( \frac{\ln (s/\mu ^2)}{(s/\mu ^2)}\right) _+ \right. \nonumber \\&\quad \left. -\, \frac{\gamma _{J\,0}^i}{2 \mu ^2} \frac{1}{(s/\mu ^2)}_+ + j_0^i\, \delta (s) \right] \nonumber \\&\quad + \left( \frac{\alpha _s(\mu )}{4\pi }\right) ^2 \left[ \frac{(\Gamma _0^i)^2}{2\mu ^2} \left( \frac{\ln ^3(s/\mu ^2)}{(s/\mu ^2)}\right) _+ \right. \nonumber \\&\quad -\,\Gamma _0^i \frac{2\beta _0 + 3\gamma _{J\,0}^i}{4\mu ^2} \left( \frac{\ln ^2 (s/\mu ^2)}{(s/\mu ^2)}\right) _+\nonumber \\&\quad + \left( j_0^i \Gamma _0^i + \frac{\beta _0 \gamma _{J\,0}^i}{2} + \frac{(\gamma _{J\,0}^i)^2}{4} - \frac{\pi ^2 (\Gamma _0^i)^2}{6} + \Gamma _1^i \right) \nonumber \\&\quad \frac{1}{\mu ^2} \left( \frac{\ln (s/\mu ^2)}{(s/\mu ^2)}\right) _+ \nonumber \\&\quad + \left( -j_0^i\left( \beta _0 + \frac{\gamma _{J\,0}^i}{2}\right) - \frac{\gamma _{J\,1}^i}{2} + \frac{\pi ^2 \gamma _{J\,0}^i \Gamma _0^i}{12} + \zeta _3 (\Gamma _0^i)^2 \right) \nonumber \\&\quad \left. \frac{1}{\mu ^2} \frac{1}{(s/\mu ^2)_+}+\, j_1^i \delta (s) \right] ,\nonumber \\&S_i(k,\mu ) = \delta (k) + \frac{\alpha _s(\mu )}{4\pi } \left[ -\frac{4\Gamma _0^i}{\mu } \left( \frac{\ln (k/\mu )}{(k/\mu )}\right) _+ \right. \nonumber \\&\quad -\,\left. \frac{\gamma _{S\,0}^i}{\mu } \left( \frac{1}{(k/\mu )}\right) _+ + s_0^i\, \delta (k) \right] \nonumber \\&\quad + \left( \frac{\alpha _s(\mu )}{4\pi }\right) ^2 \left[ \frac{8(\Gamma _0^i)^2}{\mu } \left( \frac{\ln ^3(k/\mu )}{(k/\mu )}\right) _+ \right. \nonumber \\&\quad +\, \Gamma _0^i \frac{4\beta _0 + 6\gamma _{S\,0}^i}{\mu } \left( \frac{\ln ^2(k/\mu )}{(k/\mu )}\right) _+ \nonumber \\&\quad + \left( - 4 s_0^i \Gamma _0^i + 2\beta _0 \gamma _{S\,0}^i + (\gamma _{S\,0}^i)^2 \right. \nonumber \\&\quad \left. -\, \frac{8\pi ^2 (\Gamma _0^i)^2}{3} - 4\Gamma _1^i\right) \frac{1}{\mu } \left( \frac{\ln (k/\mu )}{(k/\mu )}\right) _+ \nonumber \\&\quad + \left( -s_0^i\left( 2\beta _0 + \gamma _{S\,0}^i\right) - \gamma _{S\,1}^i - \frac{2\pi ^2 \gamma _{S\,0}^i \Gamma _0^i}{3}\right. \nonumber \\&\quad \left. \left. +\, 16\zeta _3 (\Gamma _0^i)^2 \right) \frac{1}{\mu } \frac{1}{(k/\mu )_+} + s_1^i \delta (k) \right] . \end{aligned}$$

The difference between the expressions for $C_q$ and $C_g$ is due to the additional prefactor of $\alpha _s$ in the latter. The remaining constants are given by

B2 $$\begin{aligned}&c_0^q = \left( - 8 + \frac{\pi ^2}{6}\right) C_F, \nonumber \\&c_1^q = \left( \frac{255}{8} + \frac{7\pi ^2}{2} - 30\zeta _3 - \frac{83\pi ^4}{360}\right) C_F^2 \nonumber \\&\quad + \left( -\frac{51157}{648} - \frac{337\pi ^2}{108} + \frac{313\zeta _3}{9} + \frac{11\pi ^4}{45}\right) C_F C_A \nonumber \\&\quad + \left( \frac{4085}{162} + \frac{23\pi ^2}{27} +\frac{4\zeta _3}{9}\right) C_F T_F n_f, \nonumber \\&c_0^g = \left( 5 + \frac{\pi ^2}{6}\right) C_A - 3 C_F, \nonumber \\&c_1^g = (7C_A^2 + 11C_A C_F - 6C_F \beta _0)\ln \left( \frac{-q^2-i0}{m_t^2}\right) \nonumber \\&\quad + \left( -\frac{419}{27} + \frac{7\pi ^2}{6} + \frac{\pi ^4}{72} - 44\zeta _3\right) C_A^2 \nonumber \\&\quad +\left( -\frac{217}{2} - \frac{\pi ^2}{2} + 44\zeta _3\right) C_A C_F \nonumber \\&\quad + \left( \frac{2255}{108} + \frac{5\pi ^2}{12} + \frac{23\zeta _3}{3}\right) C_A \beta _0 - \frac{5}{6}C_A T_F + \frac{27}{2} C_F^2 \nonumber \\&\quad + \left( \frac{41}{2} - 12\zeta _3\right) C_F \beta _0 -\frac{4}{3}C_F T_F + \mathcal {O}\left( \frac{q^2}{4m_t^2}\right) , \nonumber \\&j_0^q = (7-\pi ^2) C_F, \nonumber \\&j_1^q = \left( \frac{205}{8} - \frac{67\pi ^2}{6} + \frac{14\pi ^4}{15} - 18\zeta ^3 \right) C_F^2 \nonumber \\&\quad +\left( \frac{53129}{648} - \frac{208\pi ^2}{27} - \frac{17\pi ^4}{180} - \frac{206\zeta _3}{9} \right) C_F C_A \nonumber \\&\quad + \left( \frac{4057}{162} + \frac{68\pi ^2}{27} + \frac{16\zeta _3}{9}\right) C_F T_F n_f, \nonumber \\&j_0^g = \left( \frac{4}{3} - \pi ^2 \right) C_A + \frac{5}{3} \beta _0, \nonumber \\&j_1^g = \left( \frac{4255}{108} - \frac{26\pi ^2}{9} + \frac{151\pi ^4}{180} - 72\zeta ^3 \right) C_A^2 \nonumber \\&\quad -\left( \frac{115}{108} + \frac{65\pi ^2}{18} - \frac{56\zeta _3}{3}\right) C_A \beta _0 \nonumber \\&\quad - \left( \frac{25}{9}-\frac{\pi ^2}{3}\right) \beta _0^2 + \left( \frac{55}{12} - 4\zeta _3\right) \beta _0^2, \nonumber \\&s_0^q = \frac{\pi ^2}{3} C_F, \nonumber \\&s_1^q = \frac{-3\pi ^4}{10} C_F^2 + \left( \frac{-640}{27} + \frac{4\pi ^2}{3} + \frac{22\pi ^4}{45}\right) C_F C_A \nonumber \\&\quad +\left( \frac{-20}{27} - \frac{37\pi ^2}{18} + \frac{58\zeta _3}{3}\right) C_F \beta _0. \end{aligned}$$

The coefficients $s_n^g$ for the gluon soft function can directly be obtained from $s_n^q$ by replacing $C_F \rightarrow C_A$.

## Footnotes

1 Since we consider normalized distributions, there is very little dependence on the specific hard processes we consider.
